# Evidence of Blood and Muscle Redox Status Imbalance in Experimentally Induced Renal Insufficiency in a Rabbit Model

**DOI:** 10.1155/2019/8219283

**Published:** 2019-04-04

**Authors:** Konstantina P. Poulianiti, Aggeliki Karioti, Antonia Kaltsatou, Georgia I. Mitrou, Yiannis Koutedakis, Konstantinos Tepetes, Grigoris Christodoulidis, Giannis Giakas, Maria D. Maridaki, Ioannis Stefanidis, Athanasios Z. Jamurtas, Giorgos K. Sakkas, Christina Karatzaferi

**Affiliations:** ^1^Muscle Physiology & Mechanics Group, CREHP, DPESS, University of Thessaly, Trikala 42100, Greece; ^2^Human Performance Group, CREHP, DPESS, University of Thessaly, Trikala 42100, Greece; ^3^EMIP/EmPOWER, School of Health Sciences, Plymouth Marjon University, Plymouth PL6 8BH, UK; ^4^Institute for Research and Technology-CERTH, Thessaly, Trikala 42100, Greece; ^5^School of Sports, Performing Arts & Leisure, University of Wolverhampton, Wolverhampton WV1 1LY, UK; ^6^Department of Surgery, School of Medicine, University of Thessaly, Larissa 41110, Greece; ^7^DPESS, National and Kapodistrian University of Athens, Athens 17237, Daphne, Greece; ^8^Department of Nephrology, School of Medicine, University of Thessaly, Larissa 41110, Greece

## Abstract

Chronic kidney disease (CKD) is accompanied by a disturbed redox homeostasis, especially in end-stage patients, which is associated with pathological complications such as anemia, atherosclerosis, and muscle atrophy. However, limited evidence exists about redox disturbances before the end stage of CKD. Moreover, the available redox literature has not yet provided clear associations between circulating and tissue-specific (muscle) oxidative stress levels. The aim of the study was to evaluate commonly used redox status indices in the blood and in two different types of skeletal muscle (psoas, soleus) in the predialysis stages of CKD, using an animal model of renal insufficiency, and to investigate whether blood redox status indices could be reflecting the skeletal muscle redox status. Indices evaluated included reduced glutathione (GSH), oxidized glutathione (GSSG), glutathione reductase (GR), catalase (CAT), total antioxidant capacity (TAC), protein carbonyls (PC), and thiobarbituric acid reactive substances (TBARS). Results showed that blood GSH was higher in the uremic group compared to the control (17.50 ± 1.73 vs. 12.43 ± 1.01, *p* = 0.033). In both muscle types, PC levels were higher in the uremic group compared to the control (psoas: 1.086 ± 0.294 vs. 0.596 ± 0.372, soleus: 2.52 ± 0.29 vs. 0.929 ± 0.41, *p* < 0.05). The soleus had higher levels of TBARS, PC, GSH, CAT, and GR and lower TAC compared to the psoas in both groups. No significant correlations in redox status indices between the blood and skeletal muscles were found. However, in the uremic group, significant correlations between the psoas and soleus muscles in PC, GSSG, and CAT levels emerged, not present in the control. Even in the early stages of CKD, a disturbance in redox homeostasis was observed, which seemed to be muscle type-specific, while blood levels of redox indices did not seem to reflect the intramuscular condition. The above results highlight the need for further research in order to identify the key mechanisms driving the onset and progression of oxidative stress and its detrimental effects on CKD patients.

## 1. Introduction

Redox homeostasis encompasses the balance between oxidants (or prooxidants) and antioxidants and is maintained by several complex mechanisms. Redox homeostasis can be disrupted due to a dysfunction of any of these mechanisms, resulting in reactive oxygen species (ROS) and/or reactive nitrogen species (RNS) levels and/or a decreased scavenging capacity, a condition which is described as oxidative stress [1]. ROS, which will form the focus of this work, are highly reactive to proteins, membrane lipids, carbohydrates, and nucleic acids, causing irreversible damage which can affect cell survival and lead to degenerative disorders, disease, and aging [2].

Chronic kidney disease is accompanied by enhanced oxidative stress, which in turn is associated with higher risk of developing cardiovascular disease (CVD), leading to high rates of morbidity and mortality [3, 4]. Moreover, a redox imbalance in CKD is linked to pathological complications such as anemia, inflammation, malnutrition and atherosclerosis, fatigue, muscle wasting, and disuse atrophy [5–7].

Although the harmful effects of oxidative stress have been associated with the progression of CKD, its role during the different stages of the disease has not yet, been fully clarified. The majority of the existing data refer to outcomes from the advanced stages of the disease and mostly to patients undergoing hemodialysis (HD) therapy. In HD patients, four main factors have been proposed to be responsible for the redox imbalance: the uremic milieu, the HD treatment *per se*, the hemoincompatibility of dialysis system, and the concomitant drug treatment [8]. However, possible changes in redox homeostasis during the predialysis stages of CKD have not been investigated extensively probably due to the fact that CKD is “silent” at its early stages. Moreover, patients may progress to renal insufficiency as a secondary outcome of variable primary conditions, from hypertension, diabetes, nephrotic syndrome, etc. [9], conditions where monitoring oxidative stress does not figure as yet as priority of care due to insufficient evidence for clinical relevance [10]. Oxidative stress in predialysis CKD can be attributed to the cytotoxic effects of the progressive loss of renal function as well as insufficient antioxidant defense [11]. Several mechanisms link CKD and oxidative stress, such as uremic toxin-induced endothelial nitric oxide synthase (eNOS) uncoupling [12] and increased nicotinamide adenine dinucleotide phosphate-oxidases (NADPH oxidases (NOX)) activity [13]. Additionally, an emerging antioxidant deficit has been attributed mainly to dietary restrictions, diuretics use, protein energy wasting, and/or decreased intestinal absorption [14, 15].

As renal insufficiency progresses, it is also accompanied by muscular weakness and wasting, exercise intolerance, and premature fatigue, characteristics which are collectively termed as uremic myopathy [16]. Several studies have indicated that CKD is also characterized by modifications of muscle bioenergetics which also contribute to a reduction in muscle performance [17–20]. Hemodialysis treatment and/or uremia *per se* contributes to an increase in molecular oxidative damage which in turn can contribute in the deterioration of skeletal muscle functionality. This is because animal studies have shown that experimentally induced oxidative stress causes myofibrillar protein modification that promotes degradation free radical oxidation [21–23]. Moreover, the susceptibility of skeletal muscle to ROS may be differentiated depending on muscle type (fast vs. slow) [24], nutrition [25], and lifestyle habits [26]. This and other evidence collectively suggest that skeletal muscle can become extremely susceptible to degradation due to the combined effects of uremia and redox disturbances.

The aim of the current study was to evaluate possible redox disturbances in the predialysis stages of CKD, using an animal model of renal insufficiency. More specifically, we evaluated redox status indices not only in the blood but also in two different types of skeletal muscle, the fast-twitch/glycolytic psoas and the slow-twitch/oxidative soleus from the same animal donor. We also examined whether blood redox status indices could be reflecting the skeletal muscle redox status.

## 2. Material and Methods

### 2.1. Animal Model

The uremic model was based on surgical protocol modified from Gotloib et al., [22] using sham operation for the control group. All animal procedures, including surgery and euthanasia, were approved by the ethics committee of the University of Thessaly (decision 2-2/10-10-2012 and 914/10-11-2014) and the scientific committee of the University Hospital of Larisa, Greece (decision 1/4-1-2012). Animals were under veterinary care, in accordance with the National and European guidelines for the care and use of laboratory animals (EU Directive 2010/63/EU for animal experiments).

New Zealand white rabbits (female, *N* = 15) were first acclimatized to the laboratory animal unit of Medical School (University of Thessaly, Greece) for 48 hours. All animals were 5-6 months old and weighted approximately 2600 g. The animals were housed in a controlled environment with stable conditions of room temperature (RT) (22–24°C) and lighting (12 : 12 h light-dark cycle). All rabbits were fed with the same special rabbit chow containing low levels of protein (78.8 g casein “protein” per kg diet), potassium, calcium, phosphorus, and sodium (prepared by Research Diets Inc., USA; formulation code: D07122101). This was decided because key biomarkers (e.g., GSH levels) can be affected by diet [10]. Water was provided ad libitum.

After acclimatization, surgical procedures were performed (sham operation for control animals, control group, and partial nephrectomy for experimental animals, uremic group). Animals were anaesthetized by intravenous administration of a solution mixture of ketamine hydrochloride 100 mg/ml (Imalgene® 1000; Merial, Duluth, Georgia, USA) and xylazine 20 mg/ml (Rompun®; Bayer, Leverkusen, Germany), 87% and 13%, respectively (proportion 6.69 : 1 approximately). The initial dosage for the induction of anesthesia was 0.3 ml/kg body weight of the above solution mixture, i.e., Imalgene® (87%) and Rompun® (13%), intravenously (i.v.). The maintenance of anesthesia was achieved by a dose of propofol (10 mg/kg BW). Three hours before the intervention, each animal had only access to water and not to food and its weight was measured on a precision scale. Animal temperature was maintained via a heating pad.

For the uremic group, nine animals (*N* = 9) underwent removal of the left kidney after careful ligation of the left renal artery and vein and partial nephrectomy (3/4) of the right kidney. For the control group, six age-matched animals (*N* = 6) underwent sham operation. Twelve weeks after the surgery, the animals were weighed and then sacrificed by injection of sodium pentobarbital solution (50 mg/ml) which was applied in a dosage of 100 mg/kg BW followed by bilateral thoracotomy. Immediately after cardiac arrest, sample collections were done. All sample collection as well as subsequent handling and analyses were done in a blind design. It should be noted that special care was taken not to induce oxidation of glutathione, as mentioned below.

### 2.2. Blood Sampling and Treatment

Blood samples (5 ml) were collected by a heparinized syringe from rabbits' heart and aorta and were placed into ethylene diamine tetra-acetic acid- (K_2_EDTA-) containing tubes (Vacutainer Plus Plastic K_2_EDTA; Becton Dickinson). For plasma collection, blood samples were centrifuged immediately at 1370 × g for 10 min at 4°C and the supernatant was carefully collected. The remaining packed erythrocytes were lysed with 1 : 1 (*v*:*v*) distilled water, inverted vigorously, and centrifuged at 4000 × g for 15 min at 4°C. The supernatant red blood cell (RBC) lysate designated was collected. Finally, in order to obtain serum, another portion of blood sample (5 ml) was collected and placed into separate tubes containing clot activator, left for 20 min to clot at RT, centrifuged at 1370 × g, at 4°C for 10 min, and the supernatant was collected. All supernatant samples were aliquoted in Eppendorf tubes, stored at -80°C, and thawed only once before analysis.

### 2.3. Skeletal Muscle Sampling and Homogenization

Psoas and soleus muscle samples were harvested from the control (sham-operated) and uremic groups. These muscles were selected as two representative muscles, one a typical fast-twitch muscle (psoas, composed primarily of type IIX fast-twitch muscle fibers [23, 27]) and the other a typical slow-twitch muscle (soleus, composed primarily of type I slow-twitch muscle fibers). Muscle samples were frozen immediately in liquid nitrogen and stored at -80°C. For analysis, each tissue sample was thawed, excised, and kept chilled throughout homogenization. A weighted portion of each muscle was washed several times with ice-cold normal saline and was placed into prechilled tubes containing cold homogenization buffer (138 mM NaCl, 2.7 mM KCl, 1 mM EDTA, pH 7.4) and a mix of protease inhibitors (1 *μ*Μ aprotinin, 1 *μ*g/ml leupeptin, and 1 mM PMSF). Initial homogenization was achieved with an electrical homogenizer (MICCRA D-9) for 10 min with intermediate pauses of 10 s/20 s. Then an ultrasound homogenizer (UP50H) was used for 1-2 min, with intermediate pauses as before. Homogenates were filtered through four layers of medical gauze to remove connective tissue debris, incubated for 10 min at 4°C, and centrifuged at 10,000 × g for 10 min at 4°C. The supernatant homogenate was aliquoted in multiple portions and stored at -80°C for subsequent analyses.

### 2.4. Biochemical and Hematological Analyses

#### 2.4.1. Urea, Creatinine

Circulating levels of urea and creatinine were assessed, the two hallmark biomarkers that evidence renal insufficiency [28]; their concentrations in serum were determined with the colorimetric method using commercially available kits (ab83362, Abcam, United Kingdom, and ab65340, Abcam, United Kingdom), respectively, with a 96-well microtiter plate and a programmable microplate reader (Biochrom, Asys Expert 96, United Kingdom). Urea and creatinine concentrations in unknown samples were determined by comparison with the standard curves.

#### 2.4.2. Total Protein

Total protein concentration in plasma and skeletal muscle (psoas, soleus) homogenates was determined spectrophotometrically using the bicinchoninic acid (BCA) protein assay kit (Pierce, Thermo Fisher Scientific, USA). In plasma, total protein concentration was determined in order to estimate the final concentration of protein carbonyls. In muscle homogenates, total protein concentrations were determined in order to estimate the final concentrations of reduced and oxidized glutathione and protein carbonyls as well as the activities of catalase and glutathione reductase.

#### 2.4.3. Hemoglobin, Hematocrit, and Red Blood Cell (RBC) Count Analysis

CKD is associated with anemia [29]. Thus, hemoglobin, hematocrit, and red blood cell count were determined using a commercially available kit and procedures (Dutch Diagnostics BV, Zutphen, Netherlands) to examine if the model promoted the development of anemia.

### 2.5. Evaluation of Redox Status

All materials for redox marker assays were purchased from Sigma (St. Louis, MO, USA). We examined markers for antioxidant capacity, such as TAC, GSH, GR, and CAT, and markers of protein and lipid oxidation such as protein carbonyls and TBARS, respectively.

#### 2.5.1. GSH Determination

The tripeptide GSH is the most abundant nonprotein thiol and one of the main components of antioxidant capacity [30]. GSH concentration was determined in RBC lysate and skeletal muscle homogenate samples according to Rahman et al. [31], using a 96-well microtiter plate and a programmable microplate reader (Biochrom, Asys Expert 96). Briefly, samples were deproteinized with 5% trichloroacetic acid (TCA) (1 : 1 *v*/*v*) centrifuged at 16,000 × g for 10 min, and the supernatant was collected. The following reagents were added in order (all reagents in 0.1 M potassium phosphate buffer, pH 7.5, with 5 mM EDTA) to 96-well flat-bottom plate (CytoOne) in duplicate: 20 *μ*l of glutathione standard (0.103 to 26.4 *μ*Μ) or the sample to be assayed, 120 *μ*l of freshly prepared (5,5′-dithiobis 2-nitrobenzoic acid) DTNB (2 mg/3 ml), glutathione reductase (10 units), and 60 *μ*l of dihydronicotinamide-adenine dinucleotide phosphate NADPH (2 mg/3 ml) mix solution (1*v* : 1*v*). The absorbance at 415 nm was measured every 30 s, for 3 min, at RT. The rate of increase in absorbance per minute was calculated by linear regression. GSH concentration in unknown samples was determined by comparison with the standard curve.

#### 2.5.2. GSSG Determination

GSSG concentration was determined in RBC lysate and skeletal muscle homogenate samples according to Giustarini et al. [32], modified for using a 96-well microtiter plate and a programmable microplate reader. Samples were deproteinized with 5% TCA (1 : 1 *v*/*v*) centrifuged at 16,000 × g for 10 min, and the supernatant was collected. To avoid the rapid oxidation of GSH to GSSG, through the deproteination procedure, and the consequent overestimation of GSSG, the alkylating reagent N-ethylmaleimide (NEM) 310 mM was added upon collection of blood sample or the tissue homogenization. This was extracted before the actual measurement with three volumes of dichloromethane DCM, carefully collecting the upper volume of the ensuing bilayer (typically 750 *μ*l of DCM for 250 *μ*l of deproteinized supernatant), vortexed 5 min at 800 rpm at room temperature (RT), and centrifuged at 14,000 × g for 30 s at 4°C. To measure GSSG, the following reagents were added in order (all reagents in 0.1 M potassium phosphate buffer, pH 7.5, with 5 mM EDTA) to 96-well flat-bottom plate (CytoOne) in duplicate: 20 *μ*l of glutathione disulfide standard (0.103 to 26.4 *μ*Μ) or the sample to be assayed, 120 *μ*l of freshly prepared DTNB (2 mg/3 ml) and glutathione reductase (10 units) mix solution (1*v* : 1*v*), and 60 *μ*l of NADPH (2 mg/3 ml). The absorbance at 415 nm was measured every 30 s, for 3 min, at RT. The rate of increase in absorbance per minute was calculated by linear regression. GSSG concentration in unknown samples was determined by comparison with the standard curve.

#### 2.5.3. Determination of GR Activity

Glutathione reductase (GR) activity is important in the evaluation of tissue redox state but also has an antiapoptotic role [33]. GR activity was determined in RBC lysate and skeletal muscle homogenate samples according to Cribb et al. [34], using a 96-well microtiter plate and a programmable microplate reader. To measure GR activity, the following reagents were added in order (all reagents in 0.1 M sodium phosphate buffer, pH 7.5, with 1 mM EDTA) to 96-well flat-bottom plate (CytoOne) in duplicate: 150 *μ*l of 0.1 mM DTNB, 10 *μ*l of NADPH (10 mg/ml; 12 mM), and 20 *μ*l of reductase standard (0.015 to 0.50 U/ml) or the sample to be assayed. The reaction was initiated by the addition of 10 *μ*l of GSSG (1 mg/ml; 3.25 mM). For blank wells, no GSSG was added. The absorbance at 415 nm was measured every 30 s, for 3 min, at RT. The rate of increase in absorbance per minute was calculated by linear regression. GR activity in unknown samples was determined by comparison with the standard curve.

#### 2.5.4. TAC Determination

Total antioxidant capacity (TAC) is a method which is frequently used to assess the antioxidant status of biological samples and can evaluate the antioxidant response against the free radicals produced in a given condition [35]. TAC was determined in plasma and skeletal muscle homogenate samples according to Janaszewska and Bartosz [36], based on the scavenging of 2,2-diphenyl-1-picrylhydrazyl (DPPH) free radical. DPPH stock solution (10 mM) was prepared by dissolving 0.02 g DPPH in 5 ml of methanol and mix in the stirrer. The working solution was obtained by diluting the stock solution 100 times with methanol. In 20 *μ*l of plasma or homogenate, 480 *μ*l of 10 mM sodium potassium phosphate (pH 7.4) and 500 *μ*l of 0.1 mM DPPH were added and incubated in the dark for 30 min at RT. The samples were centrifuged for 3 min at 20,000 × g and 900 *μ*l of the supernatant was transferred into a clean plastic cuvette. The absorbance was read at 530 nm using a spectrophotometer. TAC values were presented as mM of DPPH reduced to 2,2-diphenyl-1picrylhydrazine (DPPH : H).

#### 2.5.5. Determination of CAT Activity

Catalase is a common enzyme found in nearly all living organisms exposed to oxygen and catalyzes the decomposition of hydrogen peroxide to water and oxygen playing a key role in protecting the cell from oxidative damage by ROS [37]. CAT activity was determined in RBC lysate and skeletal muscle homogenate samples according to Aebi [37]. 20 *μ*l of RBC lysate or homogenate was added to 2975 *μ*l of sodium potassium phosphate buffer 67 mM, pH 7.4, and the samples were incubated at 37°C for 10 min. 5 *μ*l of hydrogen peroxide 30% was added and the change in absorbance was immediately read at 240 nm for 2 min. One unit of catalase is equal to 1 *μ*mol of hydrogen peroxide H_2_O_2_ decomposed/minute. Results were normalized to hemoglobin content in the blood sample and to total protein content in muscle homogenate samples.

#### 2.5.6. PC Determination

Protein carbonylation is a type of protein oxidation that can be promoted by ROS. It usually refers to a process that forms reactive ketones or aldehydes that can be reacted by 2,4-dinitrophenylhydrazine (DNPH) to form hydrazones [38]. PC concentration was determined in plasma and skeletal muscle homogenate samples according to Fields and Dixon [39]. In 50 *μ*l of plasma or homogenate, 50 *μ*l of 20% TCA was added, incubated in the ice bath for 15 min, and centrifuged at 15,000 × g for 5 min at 4°C and the supernatant was discarded. Afterwards, 500 *μ*l of 14 mM DNPH, in 2.5 N hydrochloric acid HCl, for the sample or 500 *μ*l of 2.5 N HCl for the blank was added to the pellet. Both samples were incubated in the dark at RT for 1 h, with intermittent vortexing every 15 min. Samples were centrifuged at 15,000 × g for 5 min at 4°C. The supernatant was discarded and 1 ml of 10% TCA was added, vortexed, and centrifuged at 15,000 × g for 5 min at 4°C. The supernatant was discarded, and 1 ml of ethanol-ethyl acetate (1 : 1 *v*/*v*) was added, vortexed, and centrifuged at 15,000 × g for 5 min at 4°C. The washing step was repeated two more times. Finally, the supernatant was discarded, and 1 ml of 5 M urea (pH 2.3) was added, vortexed, and incubated at 37°C for 15 min. The samples were centrifuged at 15,000 × g for 3 min at 4°C, and the absorbance was read at 375 nm. Protein carbonyl values were obtained by using the molar extinction coefficient of DNPH (22 mM·cm^−1^).

#### 2.5.7. TBARS Determination

Lipid peroxides are oxidative degradation products of lipids with malondialdehyde (MDA) to be considered as the main marker in lipid peroxidation [40]. The TBARS assay is the simplest and most popular method for quantifying lipid peroxidation in biological samples [41]. In 100 *μ*l of plasma or homogenate, 500 *μ*l of 35% TCA and 500 *μ*l of 200 mM Tris-HCl (pH 7.4) were added and incubated at RT for 10 min. Afterwards, 1 ml of 2 M sodium sulfate Na_2_SO_4_ and 55 mM thiobarbituric acid (TBA) solution was added and incubated at 95°C for 45 min. The samples were cooled on ice for 5 min and were vortexed. 1 ml of 70% TCA was added, vortexed, and centrifuged at 15,000 × g for 3 min at 25°C. The absorbance of the supernatant was read at 530 nm. A baseline absorbance was taken into account by running a blank along with all samples during the measurement. The calculation of TBARS concentration was obtained using the molar extinction coefficient of MDA (15,600 mol/l).

#### 2.5.8. Uric Acid Determination

Uric acid is not only a by-product of purine metabolism, but it can also maintain protection against oxidative damage acting as an electron donor and scavenging peroxyl radicals, hydroxyl radicals, and singlet oxygen [42, 43]. Uric acid concentration in the serum was measured on a Clinical Chemistry Analyzer Z 1145 (Zafiropoulos Diagnostica, Athens, Greece) using commercially available kits (Zafiropoulos Diagnostica). 6 *μ*l of serum was added to 600 *μ*l of working reagent; samples were incubated for 1 min at 37°C and the absorbance was read at 340 nm.

### 2.6. Statistical Analysis

Data were analyzed using the commercially available statistical software package SPSS 22. Results were expressed as mean ± SEM and 95% confidence intervals.

Initially duplicate values were averaged before further statistical treatment. The Shapiro-Wilk test was performed to initially test whether the data were normally distributed, as it was the case.

An independent *t*-test was conducted to examine whether there were any differences in blood redox status indices between the control group and the uremic group. Similarly, an independent *t*-test was conducted to examine whether there were any differences in biochemical and hematological indices between the control group and the uremic group.

Two-way MANOVA (two groups × two muscles) was conducted to examine the effects of uremia and muscle type on muscle redox status indices. Significant interactions and main effects were further investigated using LSD post hoc analysis for multiple group comparisons.

Possible relationships between indices were examined using Pearson correlation coefficient analysis in pool and per group (control, uremic) data.

The significance level was set at *p* ≤ 0.05.

## 3. Results

Both surgery procedures (3/4 partial nephrectomy and sham operation) were well tolerated by animals as they presented with a normal after-surgery recovery. At the end of the twelve-week period postsurgery, animals' average body weight was 3728 ± 336.47 g for control and 2935 ± 288.70 g for the uremic group (*p* > 0.05).

### 3.1. Biochemical and Hematological Analyses

Renal insufficiency in the uremic group, compared to control, was reflected in the significantly raised (*p* < 0.05) blood creatinine levels (Table 1). However, urea levels were not significantly different between the groups (*p* > 0.05). Significant differences were found in the hematological profile of the uremic group compared to the control group. More specifically, hematocrit levels and RBC count were significantly lower in the uremic group compared to the control (*p* = 0.005) and (*p* = 0.001), respectively. All biochemical and hematological indices are represented in Table 1.

### 3.2. Blood Redox Status Analysis

All blood redox status indices are presented in Table 2. GSH concentration was significantly higher in the RBC of the uremic group compared to the control (*t*(9) = −2.071, *p* = 0.033). TBARS concentration tended to be higher in the plasma of the uremic group compared to the control (*p* = 0.060). No significant differences (*p* > 0.05) were found in the rest of redox status indices evaluated in the blood between the two groups. Furthermore, we found no relationship between TAC levels with hematological parameters.

### 3.3. Muscle Redox Status Analysis

No significant group main effects were found for antioxidant capacity indices (*p* > 0.05) for the two groups. Significant group main effects were found for PC concentration (*F*_(1, 21)_ = 8.902, *p* = 0.007) in both muscle types. The LSD post hoc test revealed that PC concentration was significantly higher in the uremic group compared to the control group. No group main effects were found for TBARS.

Muscle type appeared to affect the level of some indices for both the uremic and control groups. Significant muscle type main effects were found for total protein concentration (*F*_(1, 21)_ = 23.166, *p* = 0.001) with protein content of the psoas being higher than the soleus for both groups. Regarding antioxidant capacity, GSH concentration (*F*_(1, 21)_ = 6.175, *p* = 0.021) was higher in the soleus, TAC levels (*F*_(1, 21)_ = 18.316, *p* = 0.001) were higher in the psoas, and catalase activity (*F*_(1, 21)_ = 20.597, *p* = 0.001) and GR activity (*F*_(1, 21)_ = 7.498, *p* = 0.012) were higher in the soleus in both the control and uremic groups.

The LSD post hoc test revealed that total protein and TAC levels were significantly lower in the soleus compared to the psoas muscle in both the control and uremic groups. Additionally, the soleus demonstrated higher levels of TBARS and PC levels as well as higher GSH levels, catalase, and GR activities compared to the psoas muscle in both groups.

**Figure 1:**
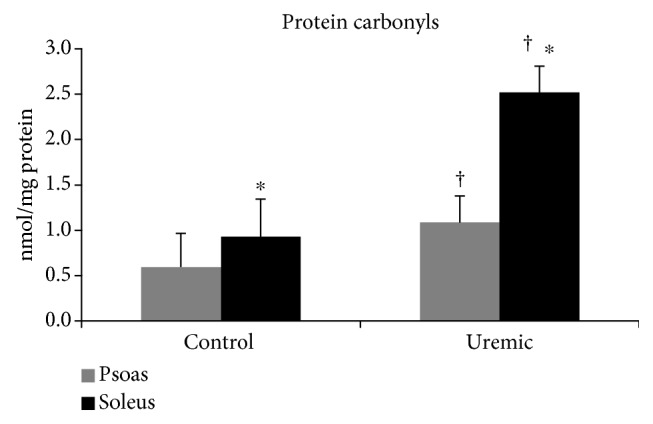
Protein carbonyl concentrations for the psoas (control: 0.596 ± 0.372 nmol/mg protein, 95% CI: lower-upper bound: 0.179-1.370; uremic: 1.086 ± 0.294 nmol/mg protein 95% CI: lower-upper bound: 0.474-1.699) and the soleus (control: 0.929 ± 0.41 nmol/mg protein, 95% CI: lower-upper bound: 0.063-1.795; uremic: 2.52 ± 0.29 nmol/mg protein, 95% CI: lower-upper bound: 1.905-3.129) in the control and uremic groups. ^∗^ depicts significant differences between the control and uremic groups; † depicts significant differences between the psoas and soleus muscles, *p* < 0.05.

Levels of PC concentration were higher in the soleus muscle in both groups (*F*_(1, 21)_ = 6.410, *p* = 0.019) (Figure 1). TBARS concentration was also higher in the soleus (*F*_(1, 21)_ = 14.703, *p* = 0.001) in both the control and uremic groups, respectively.

Finally, no interactions were found between the examined muscle types (psoas, soleus) and groups (control, uremic) for all the redox status indices (*p* > 0.05). All the results are summarized in Table 3.

### 3.4. Associations between Blood and Muscle Levels of Redox Indices

No statistically significant correlation between blood levels and muscle levels emerged for any of the antioxidant capacity and the oxidative stress indices examined in this study. This was the case either in when analysis was performed for the pool of samples or for each group separately (*p* > 0.05). Moreover, no correlations between blood levels and specific muscle type levels were observed (*p* > 0.05). Finally, no correlations were found between hematological indices and antioxidants or oxidative stress indices in the blood and skeletal muscle (*p* > 0.05).

**Figure 2:**
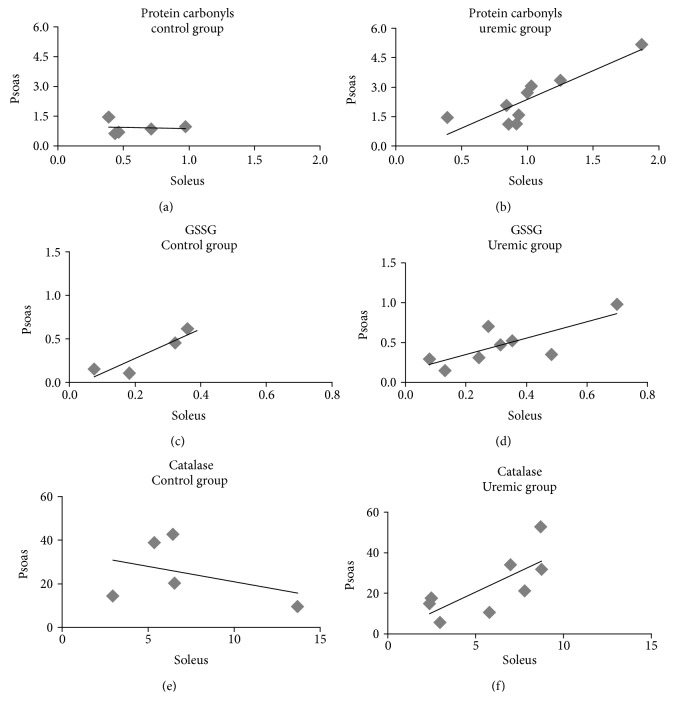
Correlation of protein carbonyl (PC) concentration (a, b), oxidized glutathione (GSSG) concentration (c, d), and catalase (CAT) activity (e, f) between the psoas and soleus muscles in the control and uremic groups, respectively. Only in the uremic group correlations were statistically significant for (b) PC levels (*r* = 0.913, *p* = 0.002), (d) GSSG levels (*r* = 0.766, *p* = 0.027), and (f) CAT activity (*r* = 0.743, *p* = 0.035).

Statistically significant correlations emerged from selective redox status indices, between the two types of skeletal muscle which however were differentiated by the group. Thus, in the uremic group, we observed strong and significant correlations in PC levels (*r* = 0.913, *p* = 0.002), in GSSG levels (*r* = 0.766, *p* = 0.027), and in CAT levels (*r* = 0.743, *p* = 0.035) between the soleus and psoas muscle samples. However, such correlations were not strong or statistically significant for the control group (Figure 2).

## 4. Discussion

We examined redox status indices in the blood and in two different types of skeletal muscle in an animal model mimicking the predialysis stage of CKD. Our main findings were that PC levels were higher in the uremic group in both muscle types and that the soleus had higher levels of TBARS, PC, GSH, CAT, and GR and lower TAC compared to the psoas in both groups. Additionally, the GSH concentration was significantly higher in the RBC of the uremic group compared to the control group. Moreover, we found no significant correlations in redox status indices between the blood and skeletal muscle.

The results demonstrated that early during the development of CKD there is an alteration in muscle redox homeostasis; importantly, both oxidative damage and antioxidant capacity seemed to be muscle type-specific. Very importantly, as it will be discussed below, we observed no association between the blood and muscle levels of the biomarkers examined, which bears significance for their prospective use in monitoring muscle pathology development.

The animal model was successfully established as it is reflected by the increases in creatinine and urea in the uremic group as compared to the control group, in agreement with observations by others using a similar model (e.g., Taes et al. [44]). Urea levels in the uremic group were almost double than those in the control group that difference was not statistically significant. This could be due to the small number of animals and animal variability. However, as a clinical observation, the doubling of urea is indicative of a considerably compromised renal function [44, 45]. Moreover, the hematological disturbances in the uremic group (significantly lower hematocrit and RBC compared to control) indicated the development of anemia, which is similar to the human condition [29].

Considering the antioxidant capacity, we observed a 40% higher GSH concentration in uremic blood samples compared to control. GSH is a primary antioxidant molecule which belongs to the endogenous defense against ROS and its role is critical for the cellular redox environment [46], since it is the most abundant nonprotein thiol that counteracts oxidative stress [47]. There are conflicting results regarding GSH concentration in CKD patients, which sometimes appear to depend on the severity of the disease and sometimes not. Thus, in moderately uremic predialysis patients, Bober et al. found higher levels of GSH compared to age-matched healthy individuals [48], in agreement to our observations. However, in other studies, lower GSH levels in the whole blood of CKD predialysis patients have been observed compared to controls [49–51] interpreted as a depletion in the antioxidant reserve. Alhamdani [52] evaluated the glutathione biosynthetic pathway in advanced uremia and hemodialysis measuring GSH levels and *γ*-glutamyl cysteine synthetase (*γ*-GCS) and glutathione synthetase (GSH-S) activities in nondialysis, hemodialysis, and continuous ambulatory peritoneal dialysis patients. Significant decreases in GSH levels and *γ*-GCS activity but not in GSH-S activity were observed in all groups of patients compared to healthy individuals. Thus, low activity of *γ*-GCS, the rate-limiting enzyme of GSH biosynthesis, may negatively affect *de novo* synthesis of GSH in those patients with low levels of GSH.

The observed increased levels of GSH concentration in uremic blood samples in our study could be attributed to an upregulation of its synthesis in response to a greater demand, especially, given the almost doubling of blood GSSG in the uremic group (levels being 1.77-fold of those of the control group). This observation, despite not reaching statistical significance, indicates a tendency for increased levels of hydrogen peroxide or lipid peroxides, similar to human studies [50]. Nonetheless, CAT also reduces hydrogen peroxide to water, but there was no statistical difference in the activity of the specific antioxidant enzyme between the two groups in our study (still, CAT activity tended to be lower in erythrocytes of the uremic group). CAT is located in peroxisomes while GSH and GPx are found mainly in the cytosol [53]. This subcellular compartmentalization is undoubtedly important for hydrogen peroxide detoxification. Based on the above, it appeared that hydrogen peroxide scavenging in circulation was undertaken to a greater degree by the glutathione redox cycling mechanism than CAT in our CKD model.

In patient studies, while general clinical guidelines are followed, dietary approaches can greatly influence GSH levels, contributing to literature's conflicting reports. GSH concentration is decreased by fasting, low-protein diets, or diets limiting in sulfur amino acids such as cysteine [54]. However, administration of *α*-lipoic acid, a naturally occurring thiol compound, increases GSH levels in several cell types and tissues [54–56] and also restores intracellular GSH in several pathological conditions [54]. Moreover, selenium (Se) as an integral part of the enzyme GPx plays a key role in GSH levels [57]. In our study, both control and uremic animals followed the same diet, carefully designed not to tax the remaining kidney function, similarly to diet guidelines followed by patients, while providing balanced nutrients and minerals, to minimize any diet-induced effect on GSH levels.

Muscle analysis per group and per muscle type indicated redox disturbances in the skeletal muscle of the uremic group. The significant increase in PC levels by approximately 1.82-fold for the psoas and 2.71-fold for the soleus in uremic compared to control psoas and soleus, respectively, points to increased levels of sarcomeric protein carbonylation. It is well established that oxidized proteins undergo diverse structure and functional changes including change in their hydrophobicity which makes them more susceptible to proteolysis [58]. Carbonylation, which is an irreversible and irreparable protein modification, tags proteins to be led through proteolysis or to form high-molecular weight aggregates through direct oxidation of side chains of lysine, arginine, proline, and threonine residues, among other amino acids. Such carbonylated aggregates can become cytotoxic and have been associated with a large number of age-related disorders [59].

Moreover, our findings are in agreement with those reported by Lim et al. [60] in the skeletal muscle of HD patients. Our results of increased PC levels in the skeletal muscle of the animal model, and the available patient evidence by Lim et al., fit with the projection of the development of muscle atrophy, which is a well-established component of uremic myopathy in end-stage hemodialysis patients [61]. Such interpretation is also corroborated by the recent finding of moderate atrophy in the psoas muscle fibers of chronically uremic animals [62]. Moreover, Lim et al. also found increased TBARS levels, for which, in agreement to the overall disease profile, we reported a tendency for increased levels in the muscle. We cannot exclude the formation of protein aggregates, but considering other evidence on progressive atrophy and the young age of the animals studied, we consider that increased PC levels are a plausible indication of atrophy mechanisms under way in predialysis stages of renal insufficiency. Taking into account the role of carbonyl stress in vascular damage [63] and the generally impaired functionality of the muscle in CKD [61, 62], early measures protecting muscle protein and vasculature from oxidation could prove of great importance for patients before progressing into the end stage where HD aggravates the redox imbalance [11] and further negative effects on muscle status accumulate.

The observed tendency for an increase in blood TBARS levels of the uremic group compared to the control group possibly revealed a predisposition towards lipid peroxidation [64], a pathogenetic factor in atherosclerosis [65]. Furthermore, lipid peroxidation negatively affects erythrocyte membrane integrity, playing a major role to their half-life shortening and thus to the development of anemia [56]. This agrees with our observation of lower RBC and Hct in our predialysis model. Papavasiliou et al. [66] found statistically significant increased TBARS levels in the plasma of predialysis patients with stages 3-5 CKD compared to healthy individuals. In the same study, patients on stages 1-2 CKD exhibited a tendency for higher TBARS levels compared to healthy individuals. In addition, stages 1-2 CKD patients exhibited significantly lower MDA levels compared to the stages 3-5 CKD patients [66]. Regarding HD patients, the large majority of studies reported increased TBARS levels in the plasma compared to healthy individuals [48, 63, 67–70], reflecting extensive lipid peroxidation, while erythropoietin treatment can help mitigate redox disturbances and inflammation, especially in the long term [70].

Taking all the above into consideration together with our findings, it could be concluded that blood lipid peroxidation in CKD emerges from the early stages of renal insufficiency.

Skeletal muscle wasting is a characteristic of several chronic diseases [71] including kidney disease. In previous work, some of us have demonstrated a fiber-type specificity of atrophy in HD patients [61, 72]. Here, we examined the redox status of the slow-twitch soleus (expressing mainly myosin heavy chain type I) and the fast-twitch psoas (expressing mainly the fast myosin heavy chain IIX) [23, 27]. Overall, the soleus muscle presented with higher levels of TBARS and PC levels but also with higher GSH levels and catalase and GR activities compared to the psoas muscle in both groups. Thus, while due to its mitochondrial content, the soleus may be expected to experience a higher oxidative stress load at the same time it appeared better equipped to withstand it, in agreement with the limited literature existing in patients [4].

One of the suggested mechanisms to explain muscle catabolism involves the tumor necrosis factor-*α* (TNF-*α*) [73, 74], via the regulation of hormones and catabolic cytokines [75–77] and can also promote muscle loss by stimulating the ubiquitin gene expression [78] and activating the nuclear factor NF-*κ*B [79]. In HD patients, Li et al. [80] observed that TNF-*α* can directly trigger protein loss and decrease in myosin heavy chain fast (MHCf) levels in the skeletal muscle. It is also known that TNF-*α*/NF-*κ*B signaling pathway is widely affected from endogenous ROS. TNF-*α* widely excites mitochondria ROS production, promoting TNF-*α*/NF-*κ*B activation [81, 82], a process which seems to be tissue-specific [80]. In line with these reports, human studies [4] indicate greater atrophy in fast-twitch muscles, perhaps via mitochondrial dysfunction in the uremic environment. Taking into consideration that fast muscle is the type mostly affected in patients [61], perhaps the redox disturbances in the psoas muscle eventually contributes to the literature observations of atrophy. On the other hand, despite the expected increased levels of mitochondrial function, the presence of higher concentrations of GSH, CAT, and GR in our study indicated that healthy soleus may have a higher antioxidant capacity than healthy psoas. In response to an augmented oxidative load due to renal insufficiency, uremic soleus appears to have further upregulated its defenses in the predialysis CKD stage, resisting the detrimental effects of ROS.

The present study evaluated the possible relationships between redox status indices in the blood and skeletal muscle under chronic renal insufficiency. Notably, we observed no correlation between the redox indices evaluated in the blood and their levels in the two different types of skeletal muscle (psoas, soleus). Correlation analysis was further expanded to explore the differences obtained in the redox markers at the blood and muscle levels in relation to parameters related to the development of anemia. However, we observed no correlation between these indices.

Although the accessibility to all tissues is feasible in animal models providing the opportunity to evaluate redox status in the tissue of interest, in humans, several difficulties and ethical limitations in such invasive processes exist, especially in patients. Thus, in the majority of human studies, redox status indices have been evaluated in the blood and results are extrapolated in tissues. However, due to limited studies [83, 84], it remains uncertain if and in which cases evaluating redox status indices in the blood adequately reflects the redox status in tissues. Rodriguez et al. [83] found that protein carbonyl concentrations were moderately (*r* = 0.51) correlated between the blood and skeletal muscle (vastus lateralis) in patients with chronic obstructive pulmonary disease. Veskoukis et al. [84] reported that four redox status indices (PC, GSH, GSSG, and catalase) in the blood adequately reflected the oxidative stress changes that happened in healthy skeletal muscle (rat gastrocnemius) after exercise and/or xanthine oxidase inhibition. This discrepancy could be explained by the differences in methodology adopted such as the type of animal model, muscle used (e.g., in Veskoukis et al. gastrocnemius, which has a different myosin composition than rabbit psoas), lab protocols, and the renal dysfunction per se having possibly an overarching systemic effect that could mask muscle's contribution to blood levels of redox indices.

If a disease state is implicated then not only the primarily suffering organic system could be contributing to a redox imbalance (as e.g., the kidney [85]) but also secondarily affected systems (e.g., muscle), as well as systemic inflammation [60, 86] and vascular stress [87] could be implicated. Measuring redox status in the blood in such a complex state may not thus allow clear conclusions with regard to tissue levels because high levels of generalized oxidative stress or increased blood antioxidant capacity could be masking the contribution of oxidative damage originating in the skeletal muscle. We cannot exclude the possibility that if uremic or control animals were exercised blood ROS levels might have reflected skeletal muscle levels. Based however on our data, in a small number of animals, with tissue samples on the resting state, we cannot presently recommend that any blood marker, among those studied, could reliably reflect intramuscular redox status.

The findings of this study should be considered in the light of some limitations. We observed statistically significant correlations in the levels of critical redox indices between the soleus and the psoas in the uremic group and not in the control, which could partly reflect the disease-induced modification of muscle properties without excluding an effect of small sample size. A bigger number of animals could better clarify the tendencies for some indices examined, perhaps also including a nonoperated control group. However, due to the high cost of the model and ethical considerations, this was not possible in the present work. While we implemented an extensive panel of redox homeostasis, based on the established laboratory expertise, future work could extend this assessment to additional redox indices and enzymes (e.g., those upstream of catalase such as superoxide dismutase (SOD)) or also consider the emerging ratio between SOD and CAT [88], the role of peroxiredoxin proteins [89]. Moreover, future work should examine the interplay between molecular mechanisms of atrophy and renal insufficiency as well as the association between a set of inflammatory biomarkers and the progression of CKD. Finally, it could be of interest to obtain data from a group of uremic rabbits also treated with antioxidant compound to evaluate the responsiveness of redox balance in the blood and in the muscle tissues.

## 5. Conclusion

In conclusion, the results of this work demonstrate that even in the predialysis stages of CKD there is an emergence of oxidative stress in the blood and a possibly adaptive response by the upregulation of the blood antioxidant defense. Moreover, carbonyl formation in both fast and slow skeletal muscle types, an indication of protein oxidation that can lead to protein degradation and proteolysis, emerges as a plausible early stimulus towards muscle atrophy observed later in advanced CKD. Last but not the least, it was found that blood levels of the redox status indices studied here did not reflect muscle concentrations. More work is needed in the direction of succeeding in less invasive monitoring of early muscle-related redox imbalances taking into consideration specific disease effects, age, and available techniques.

Our results showed redox disturbances both in the blood and muscle in an early stage of renal insufficiency, highlighting the need for further research in redox challenges imposed by chronic renal insufficiency on the skeletal muscle with a view of preserving muscle status before progression to the end stage of disease.

## Figures and Tables

**Table 1:** Biochemical and hematological indices in the control and uremic groups.

	Control group (*n* = 6)	95% confidence interval		Uremic group (*n* = 9)	95% confidence interval		*p*
		Lower bound	Upper bound		Lower bound	Upper bound	
Total protein (mg/ml)	68.59 ± 2.12	64.44	72.75	67.89 ± 2.28	63.41	72.37	0.825
Creatinine (mg/dl)	1.28 ± 0.15	1.11	1.45	2.45 ± 0.37	1.72	3.19	0.018^∗^
Urea (mg/dl)	38 ± 4.3	33.43	43.24	60 ± 11.52	37.42	82.58	0.114
Hemoglobin (mg/dl)	11.08 ± 0.92	10.33	11.81	9.80 ± 0.30	8.22	11.77	0.368
Hematocrit (%)	35.24 ± 0.79	34.54	35.93	26.06 ± 1.85	22.43	29.69	0.001^∗^
RBC (×10^6^/*μ*l)	5.10 ± 0.17	4.95	5.25	3.92 ± 0.29	3.36	4.48	0.005^∗^

Data are presented as mean ± SEM. The exact statistical significance value *p* and the 95% confidence intervals are reported.

**Table 2:** Blood redox status indices in the control and uremic groups.

Blood	Control group (*n* = 6)	95% confidence interval		Uremic group (*n* = 9)	95% confidence interval		*p*
		Lower bound	Upper bound		Lower bound	Upper bound	
Uric acid (mg/dl)	1.39 ± 0.25	0.907	1.886	1.93 ± 0.38	1.18	2.67	0.263
GSH (*μ*mol/g protein)	12.43 ± 1.01	10.448	14.412	17.50 ± 1.73	14.12	20.88	0.033^∗^
GSSG (*μ*mol/g protein)	0.027 ± 0.006	0.016	0.039	0.048 ± 0.010	0.028	0.067	0.110
Ratio (GSH/GSSG)	481 ± 53	376.357	585.642	425.28 ± 61.19	305.36	545.21	0.511
GR (U/g protein)	176.40 ± 25	127.393	225.423	153.1 ± 15.84	122.04	184.15	0.425
TAC (*μ*mol DPPH/ml)	0.786 ± 0.033	0.719	0.852	0.759 ± 0.041	0.679	0.838	0.611
CAT (U/mg protein)	342.02 ± 17.69	307.35	376.68	303.63 ± 15.63	273.01	334.26	0.131
PC (nmol/mg protein)	0.603 ± 0.09	0.427	0.780	0.620 ± 0.066	0.491	0.748	0.888
TBARS (nmol/ml)	5.12 ± 0.42	4.292	5.948	7.03 ± 0.81	5.44	8.62	0.060

Data are presented as mean ± SEM. The exact statistical significance value *p* and the 95% confidence intervals are reported. GSH: reduced glutathione; GSSG: oxidized glutathione; TAC: total antioxidant capacity; CAT: catalase; PC: protein carbonyls; TBARS: thiobarbituric acid reactive substances, ^∗^ statistical significance between the control and uremic groups, *p* < 0.05.

**Table 3:** Psoas and soleus muscle biochemical and redox status indices in the control and uremic groups.

	Psoas muscle						Soleus muscle					
	Control group (*n* = 5)^≠^	95% confidence interval		Uremic group (*n* = 8)^≠^	95% confidence interval		Control group (*n* = 5)^≠^	95% confidence interval		Uremic group (*n* = 8)^≠^	95% confidence interval	
		Lower bound	Upper bound		Lower bound	Upper bound		Lower bound	Upper bound		Lower bound	Upper bound
Total protein (mg/ml)	5.753 ± 0.43	4.865	6.642	4.537 ± 0.34	3.836	5.240	3.155 ± 0.478^†^	2.161	4.148	3.29 ± 0.33^#^	2.588	3.992
GSH (*μ*mol/g protein)	5.539 ± 1.69	2.014	9.065	6.087 ± 1.34	3.300	8.875	8.09 ± 1.89^†^	4.155	12.039	11.41 ± 1.34^#^	8.625	14.20
GSSG (*μ*mol/g protein)	0.265 ± 0.09	0.060	0.470	0.322 ± 0.07	0.160	0.484	0.331 ± 0.11	0.102	0.560	0.472 ± 0.78	0.310	0.634
Ratio (GSH/GSSG)	18.66 ± 11.11	-4.455	41.775	25.60 ± 8.787	7.326	43.874	46.62 ± 12.43	20.782	72.468	33.61 ± 8.79	15.34	51.89
GR (U/g protein)	10.59 ± 2.25	5.912	15.273	12.72 ± 1.78	9.018	16.419	18.41 ± 2.52^†^	13.184	23.651	16.42 ± 1.78^#^	12.724	20.125
TAC (*μ*mol DPPH/ml)	0.595 ± 0.06	0.458	0.732	0.519 ± 0.05	0.411	0.627	0.322 ± 0.07^†^	0.169	0.476	0.264 ± 0.05^#^	0.155	0.372
CAT (U/mg protein)	6.992 ± 4.71	-2.799	16.785	5.721 ± 3.72	-2.020	13.463	29.09 ± 5.26^†^	18.140	40.063	23.60 ± 3.72^#^	15.863	31.346
TBARS (nmol/ml)	3.179 ± 1.06	0.983	5.376	2.297 ± 0.835	0.561	4.034	6.81 ± 1.18^†^	4.350	9.262	6.25 ± 0.83^#^	4.511	7.984

Data are presented as mean ± SEM. Confidence intervals are reported. TBARS: thiobarbituric acid reactive substances; GSH: reduced glutathione; GSSG: glutathione oxidized; TAC: total antioxidant capacity; CAT: catalase. ^†^Statistical significance between control psoas and control soleus, ^#^statistical significance between uremic psoas and uremic soleus, *p* < 0.05. ^≠^It should be noted that muscle analysis refers to *n* = 5 for the control and *n* = 8 for the uremic group as a batch of samples became inappropriate for analysis.

## Data Availability

The data used to support the findings of this study are available from the corresponding author upon request.

## Abbreviations

BCA: Bicinchoninic acid
BW: Body weight
CKD: Chronic kidney disease
CVD: Cardiovascular disease
DCM: Dichloromethane
DNPH: 2,4-Dinitrophenylhydrazine
DPPH: 2,2-Diphenyl-1-picrylhydrazyl
DTNB: 5,5′-Dithiobis 2-nitrobenzoic acid
EDTA: Ethylenediaminetetraacetic acid
*γ*-GCS: *γ*-Glutamyl cysteine synthetase
GPx: Glutathione peroxidase
GSH: Reduced glutathione
GSH-S: Glutathione synthetase
GSSG: Oxidized glutathione
GR: Glutathione reductase
HCl: Hydrochloric acid
HD: Hemodialysis
H_2_O_2_: Hydrogen peroxide
KCl: Potassium chloride
K_2_EDTA: Ethylene diamine tetra-acetic acid
MDA: Malondialdehyde
MHCf: Myosin heavy chain fast
NaCl: Sodium chloride
NADPH: Dihydronicotinamide-adenine dinucleotide phosphate
Na_2_SO_4_: Sodium sulfate
NEM: N-Ethylmaleimide
NF-*κ*B: Nuclear factor *κ*Β
ox-LDL: Oxidatively modified low-density lipoprotein
PC: Protein carbonyls
PMSF: Phenylmethylsulfonyl fluoride
RBC: Red blood cell
ROS: Reactive oxygen species
RT: Room temperature
SEM: Standard error of the mean
SOD: Superoxide dismutase
TAC: Total antioxidant capacity
TBA: Thiobarbituric acid
TBARS: Thiobarbituric acid reactive substances
TCA: Trichloroacetic acid
t-GSH: Total glutathione
TNF-a: Tumor necrosis factor-*α*.
